# Supplementary material for: An early warning signal for grassland degradation on the Qinghai-Tibetan Plateau
Source: Nat Commun. 2023 Oct 12;14:6406. doi: 10.1038/s41467-023-42099-4 (PMC10570289; doi:10.1038/s41467-023-42099-4)
Supplement: Supplementary file 1 — Supplementary Information [file 41467_2023_42099_MOESM1_ESM.pdf]

SUPPLEMENTARY INFORMATION

An early warning signal for grassland degradation on the Qinghai-Tibetan Plateau

Qiuan ZHU[1*#], Huai CHEN[2#], Changhui PENG[3,4], Jinxun LIU[5], Shilong PIAO[6], Jin-Sheng HE[7], Shiping WANG[6], Xinquan ZHAO[8], Jiang ZHANG[1], Xiuqin FANG[1], Jiaxin JIN[1], Qi-En YANG[8], Liliang REN[9], Yanfen WANG[10*]

SUPPLEMENTARY INFORMATION CONTENTS:

1. Supplementary Note 1.

Effects of climate change, elevated CO_2 concentration and stocking rate on net primary productivity of QTP grasslands.

2. Supplementary Note 2.

Short review on degradation and overgrazing status of grassland on the QTP.

3. Supplementary Figures.

Supplementary Figures 1 - 9

4. Supplementary Tables.

Supplementary Table 1 - 5

5. Supplementary References

1. Supplementary Note 1

Effects of climate change, elevated CO_2 concentration and stocking rate on net primary productivity (NPP) of QTP grasslands

To evaluate the effects of climate change and elevated CO_2 concentration on grassland net primary productivity (NPP) on the QTP, we conducted three simulations for the period 1980-2017 (Table SN1.1). The baseline simulation (S01) was forced with constant climate and CO_2 concentration condition in 1980 without grazing activities. The other two simulations in which the grazing activity were initialized in 1980 and the stocking rate was allowed to vary annually. In one simulation (S02), climate and CO_2 conditions remained constant as in 1980. In the other simulation (S03), actual annual climate conditions and CO_2 levels between 1980 and 2017 were used to force the model.

The differences of annual mean (from 2016 to 2017) NPP of grassland on the QTP were calculated between different scenarios. Figure SN1.1a showed the spatial patterns of combined effects of climate change, elevated CO_2 and grazing on grassland NPP. The positive values in Figure SN1.1a indicated the region in which the positive effects of climate change and elevated CO_2 can totally offset the negative effects of grazing on grassland NPP. NPP increased to a much greater extent and over a larger area, reflecting the beneficial effects of climate change and elevated CO_2 (Figure SN1.1a). The exception was overgrazed areas, where the positive effects of climate change and elevated CO_2 cannot compensate for the negative effects of grazing on grassland NPP (Figure SN1.1a). Figure SN1.1b showed the solo effects of grazing on grassland NPP on the QTP. The grazing activities indicated net negative effects across the whole grassland area, particularly in overgrazed areas. Then we can identify the regions in which whether positive effects of climate change and elevated CO_2 can offset the negative effects of grazing (Figure SN1.1c).

Table SN1.1 Simulation scenarios set for evaluating the effects of climate change, elevated CO_2 concentration and stocking rate on net primary productivity of QTP grasslands

Scenario	Simulation for the period 1980-2017	
	climate change and CO_2 concentration	Stocking rate
S01	constant as in 1980	None
S02	constant as in 1980	varied annually
S03	variable climate conditions and CO_2 concentration through 1980-2017	varied annually

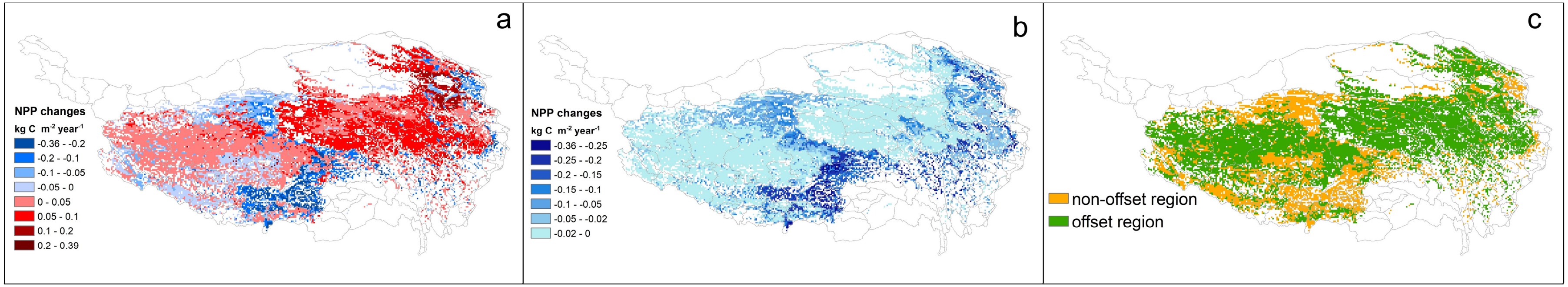

Figure SN1.1 Discrepancy of mean net primary productivity (NPP) (mean of years 2016 to 2017) of grasslands on the Qinghai-Tibetan Plateau between different scenarios list in Table SN1.1: a. S03 minus S01 (combined effects of climate change, elevated CO_2 and grazing); b. S02 minus S01 (effects of grazing only); c. distribution of regions in which whether positive effects of climate change and elevated CO_2 offset the negative effects of grazing. The maps were masked with an initial grassland distribution based on 1:1000000 China vegetation map.

2. Supplementary Note 2

Short review on degradation and overgrazing status of grassland on the QTP

2.1 Grassland degradation status on the QTP

Although many experimental studies conducted at site or local scale have evaluated the effects of grazing on grassland structure, soil qualities and other parameters related to productivity, few studies have simultaneously investigated the status of grassland degradation and grazing activities across the entire QTP.

For example, although many previous reports showed the percentages of degraded grasslands on QTP [1, 2, 3, 4, 5, 6, 7, 8, 9, 10], the mentioned percentages were simply taken from earlier work that did not undergo peer review. To cite one instance: Cao et al. (2019)[11] reported that different studies have concluded different extents of QTP grassland degradation, yet we found that most of those studies simply declared high rates of degradation on alpine grasslands on the QTP without directly analyzing it [1, 2, 6, 8, 10, 12, 13]. Most of the studies were carried out at site or local level and there is also no specific information on the relationship between grassland degradation and stocking rate over the entire QTP.

One revealing example of this problem is the claim by Cao et al. (2019)[11] that 90% of QTP grasslands have been degraded to at least some extent, for which the work of Harris (2010)[14] was cited. We found that this figure actually refers to the percentage of grassland degradation across all of China, not specifically on the QTP [14, 15, 16, 17, 18, 19, 20, 21]. This figure was also mentioned in a monograph by Lu et al. (2006) [22], which was then cited by many of the abovementioned or following studies. Wang et al. (2015)[23] suggested that the figure of 90% is likely to include many areas showing slight, temporary degradation due to short-term variations in precipitation, temperature, and other random factors. Indeed, one NPP-based assessment of grassland degradation concluded that 22.7% of total grassland area in China was in a degraded state between 1982 and 2010 [24]. The figure of 90% is no longer cited in the *National Grassland Monitoring Report* (www.forestdata.cn/index.html) or the report *Forest and Grass Resources and Ecological Status in China* (2021).

Our review of the literature (Table SN2.1) indicates that the reported extent of degradation across the entire QTP has ranged from 21% to 40% from the 1980s to 2000s, while the extent in Qinghai Province has ranged from 17% to 39% from the 1980s to 1990s, and the extent in Tibet Province has ranged from 13% to 30% from the 1980s to 1990s. Most of the previous studies that we examined did not report how these percentages were calculated, and none analyzed stocking rate. Some studies have used remote sensing data to model or made literatures reviewing the status of grassland degradation across the Plateau [12, 13, 25], concluding that 32-39% of QTP grasslands have been degraded. However, those studies did not examine how much stocking rate has contributed to that degradation.

Some studies have analyzed grassland degradation on a global scale, thereby including the QTP without focusing on it. Such work may provide insights relevant for the QTP. Kwon et al. (2016)[26] developed a modeling framework to quantify the global costs of grassland degradation in terms of inaction and loss of livestock productivity. Bardgett et al. (2021)[27], although they cite the potentially misleading figure of 90% degradation on the QTP from Cao et al. (2019)[11], mapped global grassland degradation based on greenness data derived from Normalized Difference Vegetation Index (NDVI). Gang et al. (2014)[28] assessed global patterns of grassland degradation from 2000 to 2010 using NPP. While that work suggested that degradation has increased over globe, comparing it with our study is difficult since the authors did not report any data specifically for the Plateau. Ultimately, the relevance of global grassland studies for the QTP is limited given the wide variation in extent of grassland degradation around the world, which in one study varied from 1 billion to over 6 billion hectares, with equally wide disagreement in the spatial distribution of the degradation [29]. In addition, none of these global studies mentioned above analyzed stocking rate.

Table SN2.1 Collected grassland degradation information from literatures (NA: Not Available)

Studies	Grassland degradation declared Percentage (Based area)	Period	Indicator	Methods	Related stocking rate information	Reference
Ma et al. (1999)[5]	33% (QTP)	1980s~1990s	NA	NA	NA	NA
World Bank Group (2001)[21]	30% (Qinghai) & 26% (Tibet)	NA	NA	NA	NA	Ministry of Agriculture of China
Sheehy (2001)[7]	29% (Qinghai) & 18% (Tibet)<br>31% (Qinghai) & 30% (Tibet)<br>21% (QTP)<br>33% (QTP)	1980s<br>1990s<br>1980s<br>1990s	NA	NA	NA	NA
Berry (2003)[15]	30% (Qinghai) & 26% (Tibet)	NA	NA	NA	NA	Ministry of Agriculture of China
Hu and Zhang (2003)[16]	17% (Qinghai) & 14% (Tibet)<br>39% (Qinghai) & 15% (Tibet)	1990<br>1999	NA	NA	NA	NA
Wang et al. (2004)[20]	30% (Qinghai) & 26% (Tibet)	NA	NA	NA	NA	Ministry of Agriculture of China
Lu et al. (2006)[22]	17.0% (Qinghai) & 13.9% (Tibet)	~1990	NA	NA	NA	NA
He et al. (2008)[30]	21.4% (QTP)<br>32.7% (QTP) (1990s)	1980s<br>1990s	NA	NA	NA	NA
Liu et al. (2008)[4]	36.12% (Three-River Headwaters)	1990s	Grassland coverage gradient	Remote sensing data analysis	NA	-
Feng et al. (2010)[3]	33% (QTP)	1980s~1990s	NA	NA	NA	Ma et al. (1999)[5]
Li et al. (2013)[12]	33% (QTP)	1990s	NA	NA	NA	Sheehy (2001)[7]
Ren et al. (2013)[6]	33% (QTP)	1980s~1990s	NA	NA	NA	Feng et al. (2010)[3]
Cai et al. (2015)[1]*	~40% (QTP)	1990s	NA	NA	NA	Liu et al. (2008)[4]
Wang et al. (2016)[13]	38.8% (QTP)	2000s	NPP	remote sensing data based model (CASA)	NA	-
Zhou et al. (2023)[25]**	32.69% (QTP)	1990s	NA	NA	NA	Foggin et al. (1996)[31]

*note: Actually, the study of Liu et al. (2008)[4] was conducted only over Three-River Headwaters based on remote sensing data, and found that the 36.12% of grassland in this area was degraded between 1990s and 2004.

**note: Did not find the specific percentage number in the study of Foggin et al. (1996)[31]

2.2 Comparison of simulated and data derived stocking rate on the QTP

Based on NPP data and county scale of sheep number data in year 2000 and 2010, Sun et al. (2022)[32] extrapolated actual stocking rate maps of QTP for years of 1990,1995,2000,2005,2010 and 2015. We compared our simulated multi-year mean stocking rates to that reported in the study of Sun et al. (2022)[32].

The range of 0-1.1 SU ha^{-1} $year^{-1}$ in the Sun's study (Figure SN2.1a) is lower than our simulated stocking rates, especially in the eastern and southern regions of the QTP (Figure SN2.1b). The difference of stocking rate between Sun's study and our results are mainly within the range between 0.0 to 2.0 SU ha^{-1} $year^{-1}$ (61.3% area percentage, Figure SN2.1b). We suspect, based on our extensive literature review, that the Sun's study underestimates stocking rate on the QTP, particularly in the eastern and southern areas, such that stocking rate lies below the threshold across the entire Plateau (Figure SN2.1c).

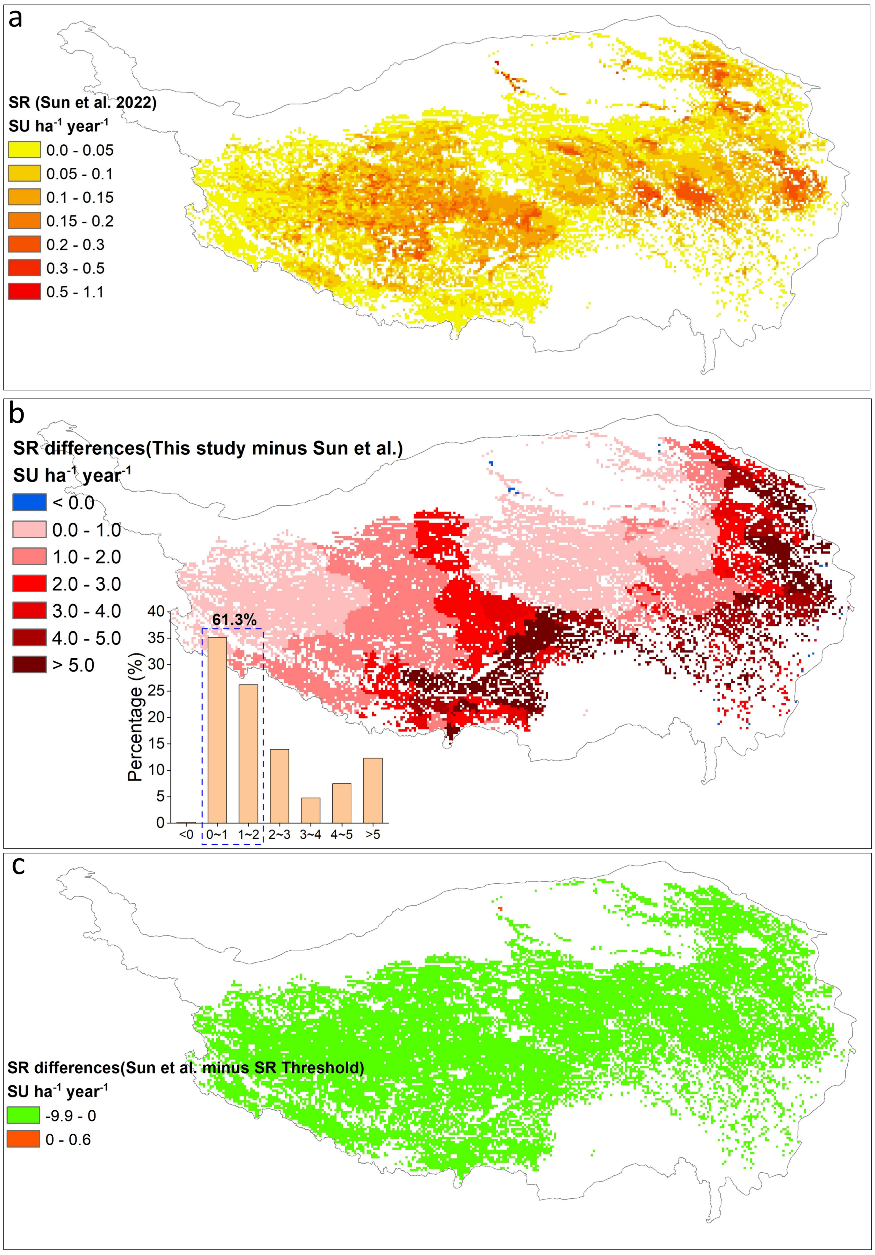

Figure SN2.1 a. Stocking rate map extracted from Sun et al. (2022)[32]; b. Stocking rate differences between calculated in this study and that in Sun et al. (2022)[32]; c. Comparison between stocking rate calculated in Sun et al. (2022)[32] and stocking rate threshold created in this study. Maps were masked with an initial grassland distribution based on 1:1000000 China vegetation map. SR: stocking rate. SU, sheep unit.

Our estimated stocking rates were slightly higher, by 1.0- 3.0 SU ha^{-1} $year^{-1}$ (69.5% area percentage), than experimentally determined stocking rates reported in another study[33], where stocking rates ranged from 0 to 13.6 SU ha^{-1} $year^{-1}$ and high stocking rates occurred mostly in the eastern and southern regions of the QTP (Figure SN2.2). Based on those data, stocking rate across 98% of QTP grasslands lies below the threshold (Figure SN2.2c). We also made a comparison between Sun's and Meng's studies, the stocking rate created by Sun et al. is far below that generated by Meng et al. (Figure SN2.2d).

These considerations highlight how much estimates of stocking rate and therefore the current extent of grassland degradation can differ. Nevertheless, this variability should not influence the estimation and reliability of our stocking rate thresholds.

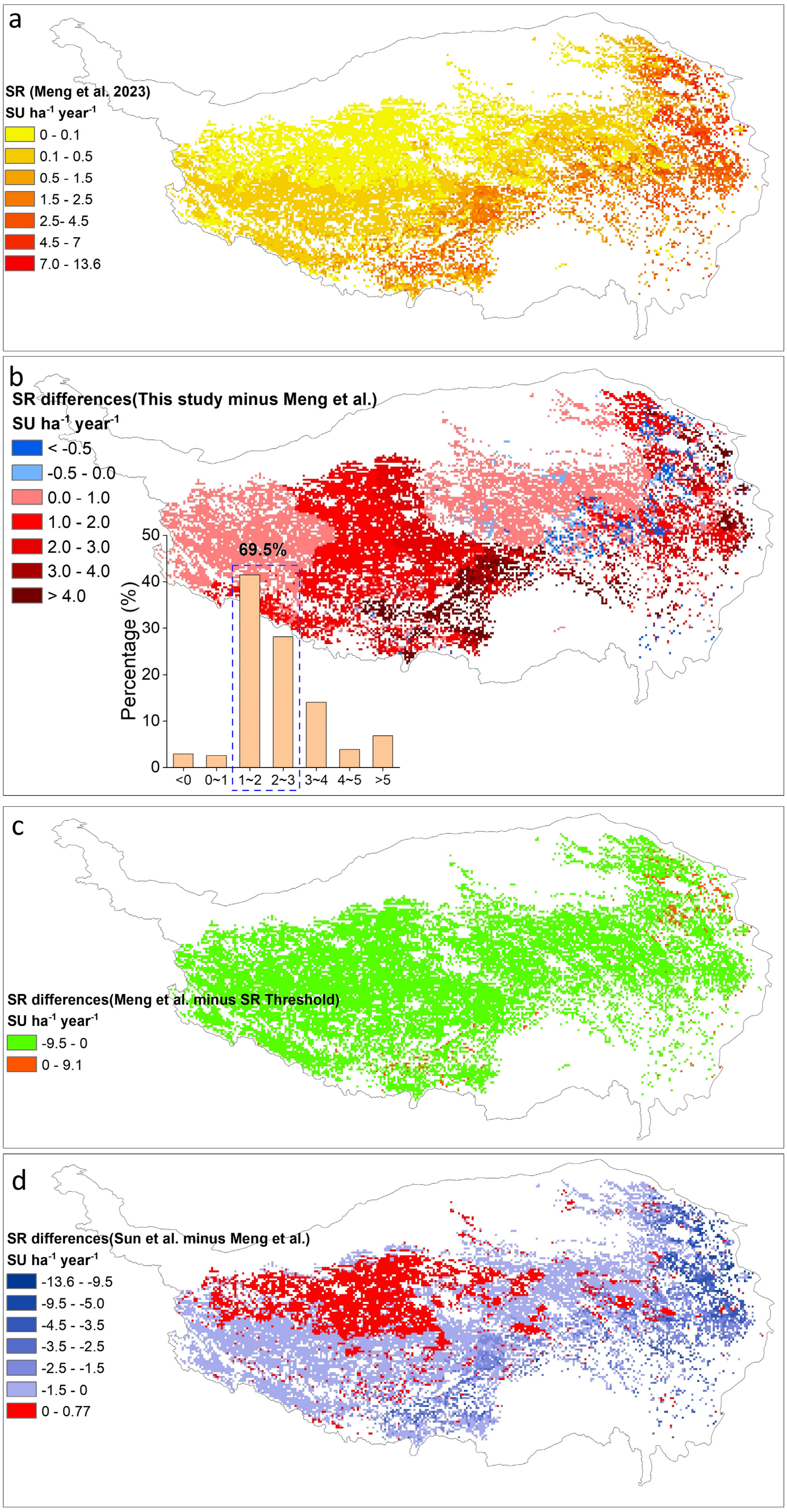

Figure SN2.2 a. Stocking rate map extracted from Meng et al. (2023)[33]; b. Stocking rate differences between calculated in this study and that in Meng et al. (2023)[33]; c. Comparison between stocking rate calculated in Meng et al. (2023)[33] and stocking rate threshold created in this study; d. Comparison between stocking rate calculated in Sun

et al. (2022)[32] and that in Meng et al. (2023)[33]. Maps were masked with an initial grassland distribution based on 1:1000000 China vegetation map. SR: stocking rate. SU, sheep unit.

2.3 Extent of overgrazing on QTP grasslands

In our study, the overgrazing status is defined as stocking rate lager than the stocking rate threshold. Our results showed that the overgrazing area percentage was about 20%. Since our defined stocking rate at threshold level would induce an extreme grassland degradation, our results figured the most serious overgrazing status to some extent.

Overgrazing rates have been reported to be 28.6% across the entire QTP in the 1990s [34], 16% for Qinghai Province in 2003-2004 and 78% for Tibet Province during the same period [35]. Zhang et al. (2014)[36] reported rates of 27-89% during 1996-2008, based in part on the abovementioned studies of Yang et al. (2000)[34] and Qian et al. (2007)[35]. Based on the annual *Supervision Report of National Grasslands* from the Ministry of Agriculture of China, mean overgrazing rates were 16% for Qinghai and 24% for Tibet for the period 2010-2017, with the annual overgrazing rate declining from 39% to 9.8% in Qinghai province and from 40% to 13% in Tibet between 2006 and 2017 (Table SN2.2). Unfortunately, the annual reports did not specify how those figures were calculated. Overgrazing may be quite heterogeneous on the Plateau.

Table SN2.2 Collected grassland overgrazing information from literatures (NA: Not Available)

Studies		Overgrazing rate declared Percentage (Based area)	Period	Methods	Reference
Yang and Yang 2000[34]		28.6% (QTP)	1990s	Based on grassland potential productivity calculated using Miami model	-
Qian et al. 2007[35]		16% (Qinghai) & 78% (Tibet)	2003~2004	Based on grassland potential productivity calculated based on NDVI data	-
He et al. 2008[30]		1.25~102.87% (Different regions across QTP)	1980s~1990s	NA	NA
Zhang et al. 2014[36]		27~89% (Different regions across QTP)	1996~2008	NA	Yang and Yang 2000[34] Qian et al. 2007[35]
Supervision report of national grasslands	2006	39% (Qinghai) & 38% (Tibet)	2006	NA	Ministry of Agriculture of People's Republic of China (Report could be obtained from http://www.moa.gov.cn/ or http://www.forestdata.cn/index.html)
	2007	38% (Qinghai) & 40% (Tibet)	2007	NA	
	2008	37% (Qinghai) & 38% (Tibet)	2008	NA	
	2009	26% (Qinghai) & 39% (Tibet)	2009	NA	
	2010	25% (Qinghai) & 38% (Tibet)	2010	NA	
	2011	25% (Qinghai) & 32% (Tibet)	2011	NA	
	2012	16% (Qinghai) & 29% (Tibet)	2012	NA	
	2013	14% (Qinghai) & 22% (Tibet)	2013	NA	
	2014	13% (Qinghai) & 19% (Tibet)	2014	NA	
	2015	13% (Qinghai) & 19% (Tibet)	2015	NA	
	2016	11.9% (Qinghai) & 16% (Tibet)	2016	NA	
	2017	9.8% (Qinghai) & 13% (Tibet)	2017	NA	

3. Supplementary Figures

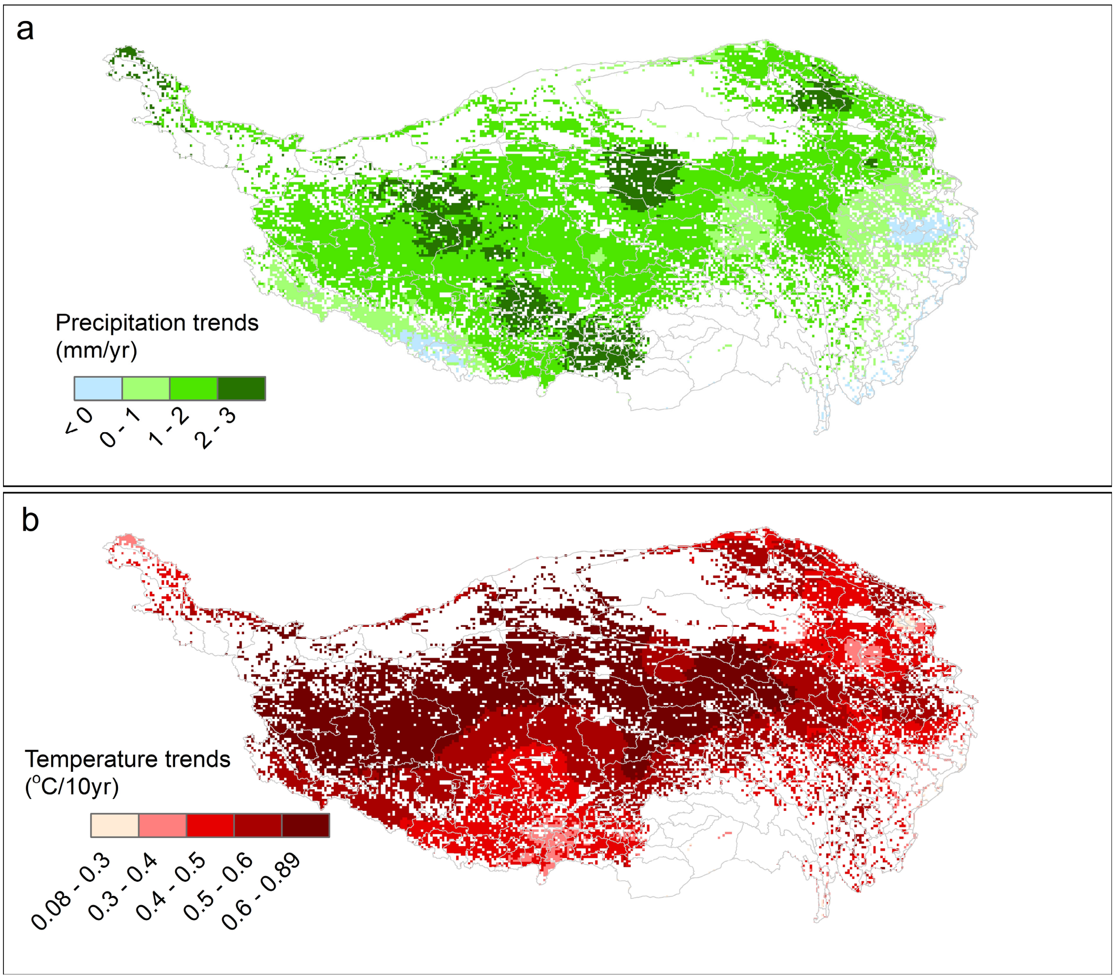

Supplementary Figure 1 Linear trends of precipitation (a) and temperature (b) over QTP grasslands for the period 1980-2017 based on gridded model forcing data of precipitation and temperature. Maps were masked with an initial grassland distribution based on 1:1000000 China vegetation map.

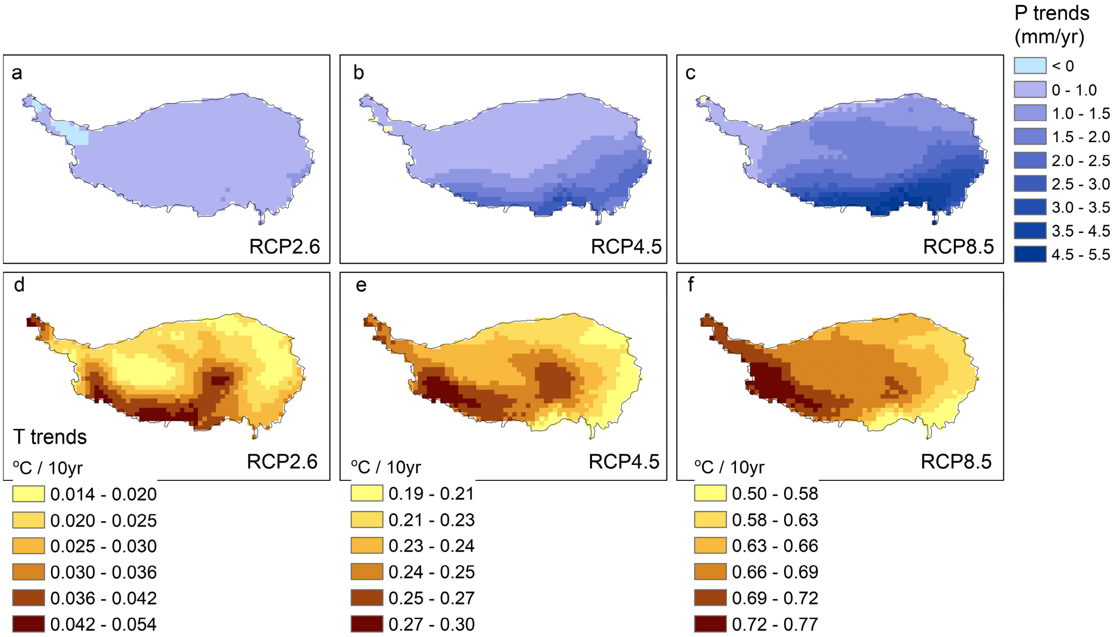

Supplementary Figure 2 Linear trends of precipitation (P) (a, b, c) and temperature (T) (d, e, f) over the QTP for the period 2020-2100 under three Representative Concentration Pathways (RCP2.6, 4.5 and 8.5) based on mean annual precipitation and temperature from the multi-model, developed from 24 General Circulation Models (GCMs, see Supplementary Table 5).

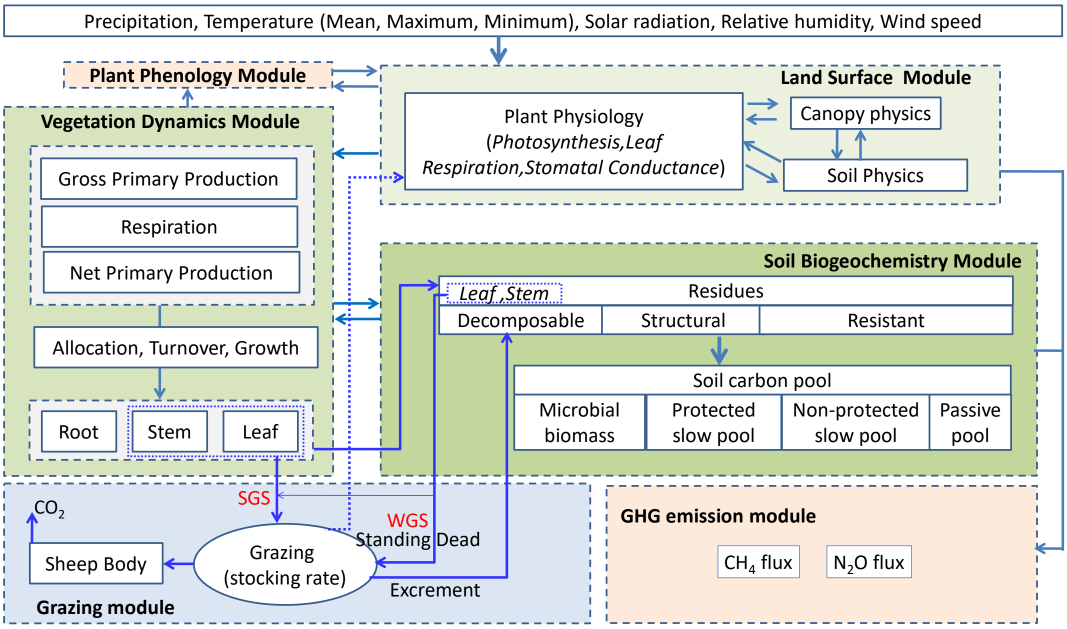

Supplementary Figure 3 Schematic illustrating the integration of grazing process (light blue box) into the TRIPLEX-GHG model. SGS, summer grazing season; WGS, winter grazing season.

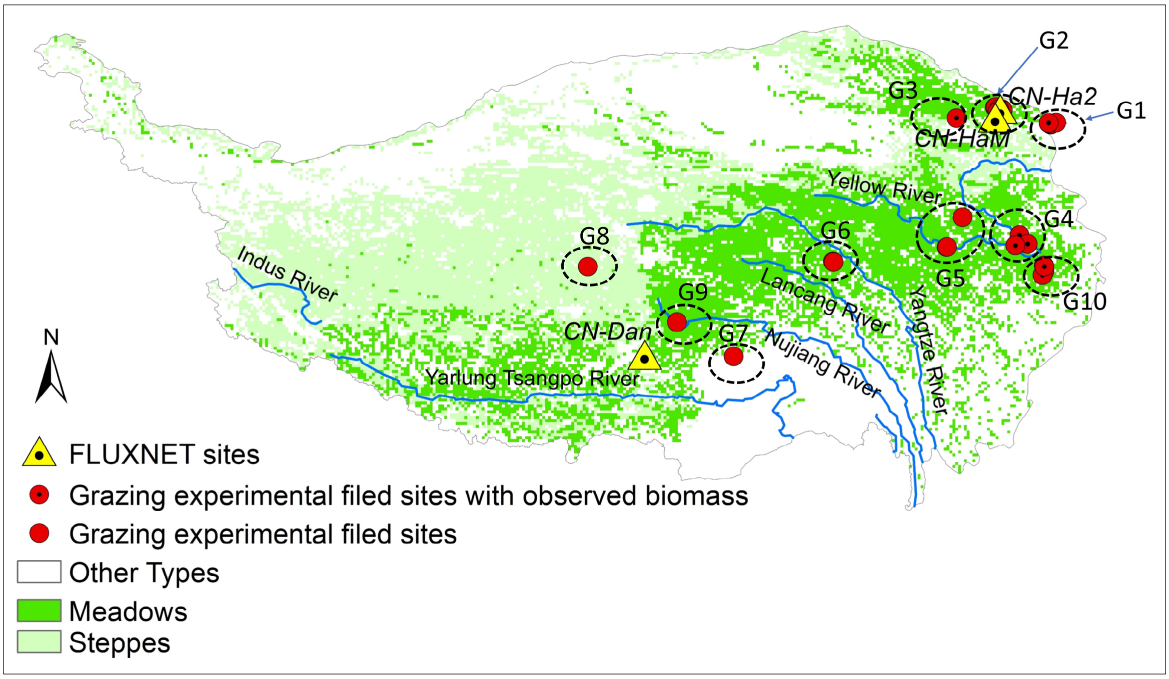

Supplementary Figure 4 Geographical distribution of sites used to evaluate model performance. Experimental field sites where a gradient of stocking rate was designed are shown as red circles, if only experimental stocking rate was used; red circles with a black dot in the center, if biomass data were also used; or as triangles, if FLUXNET data on gross primary productivity (GPP) were also used (https://fluxnet.org/sites/site-list-and-pages/). Experimental field sites were grouped at the county level into clusters G1 to G10 (see Supplementary Table 3). The map was masked with an initial grassland distribution based on 1:1000000 China vegetation map.

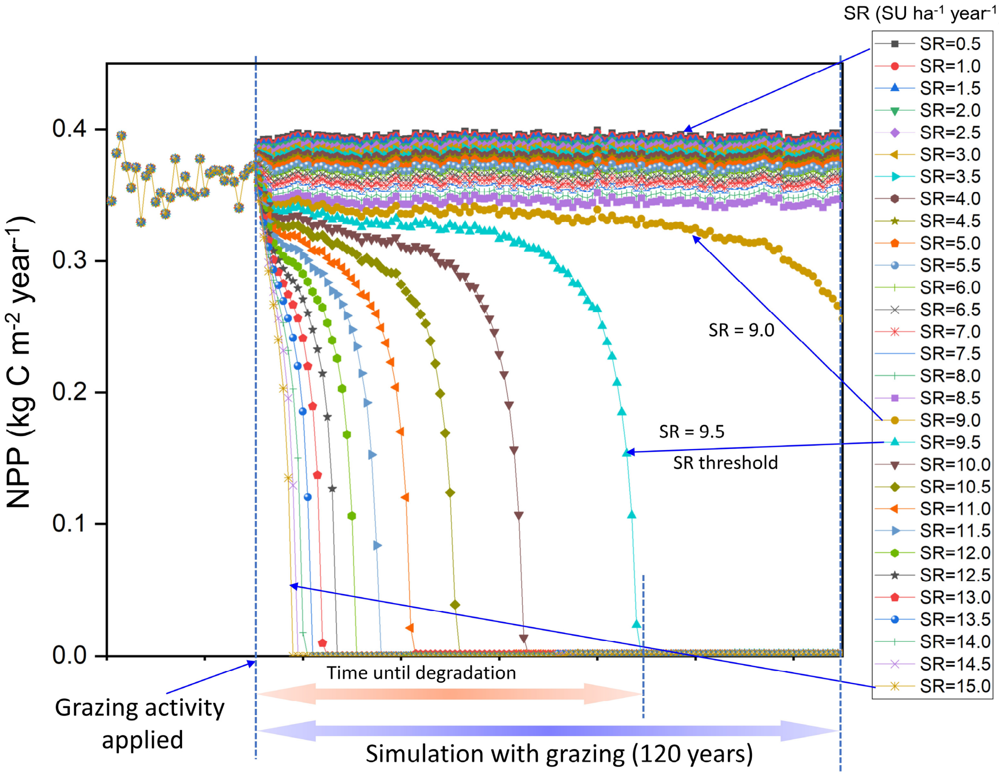

Supplementary Figure 5 Schematic of how changes in net primary productivity (NPP) of grasslands subject to different stocking rates can be used to determine "stocking rate threshold" and "time until degradation". The process assumed that grassland productivity would decrease as stocking rate increased. The model was run under different stocking rates, from non-grazing (stocking rate = 0.0 SU ha^{-1} year^{-1}) to maximum possible grazing (stocking rate = 15 SU ha^{-1} year^{-1}), increasing in a step of 0.5 SU ha^{-1} year^{-1} for a period of 120 years. When NPP fell to 1% of the original NPP before application of the stocking rate, that stocking rate was defined as the stocking rate threshold. The interval between onset of stocking rate and when NPP fell to 1% of the original NPP was defined as the time until degradation. SR: stocking rate. SU, sheep unit.

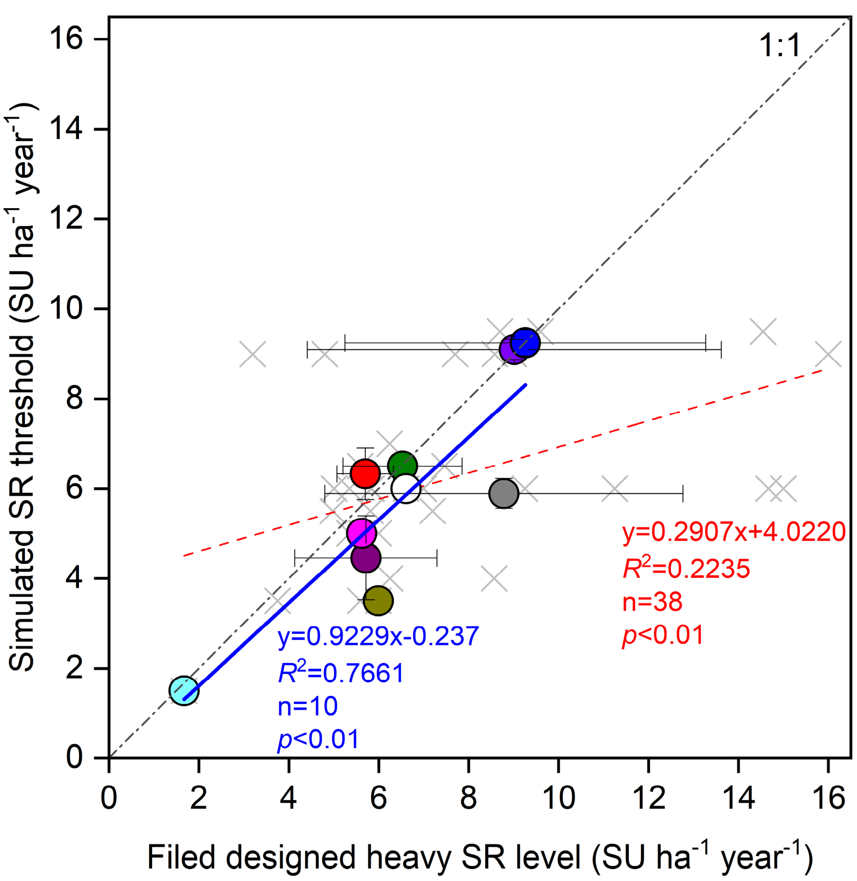

Supplementary Figure 6 Comparison between designed heavy stocking rates at experimental field sites and corresponding simulated stocking rate thresholds in this study. The red dashed line represents the linear regression across all sites; the blue line, linear regression across ten county clusters (see Supplementary Table 3 and Supplementary Figure 4). Error bars represent standard deviation. SR: stocking rate. SU, sheep unit.

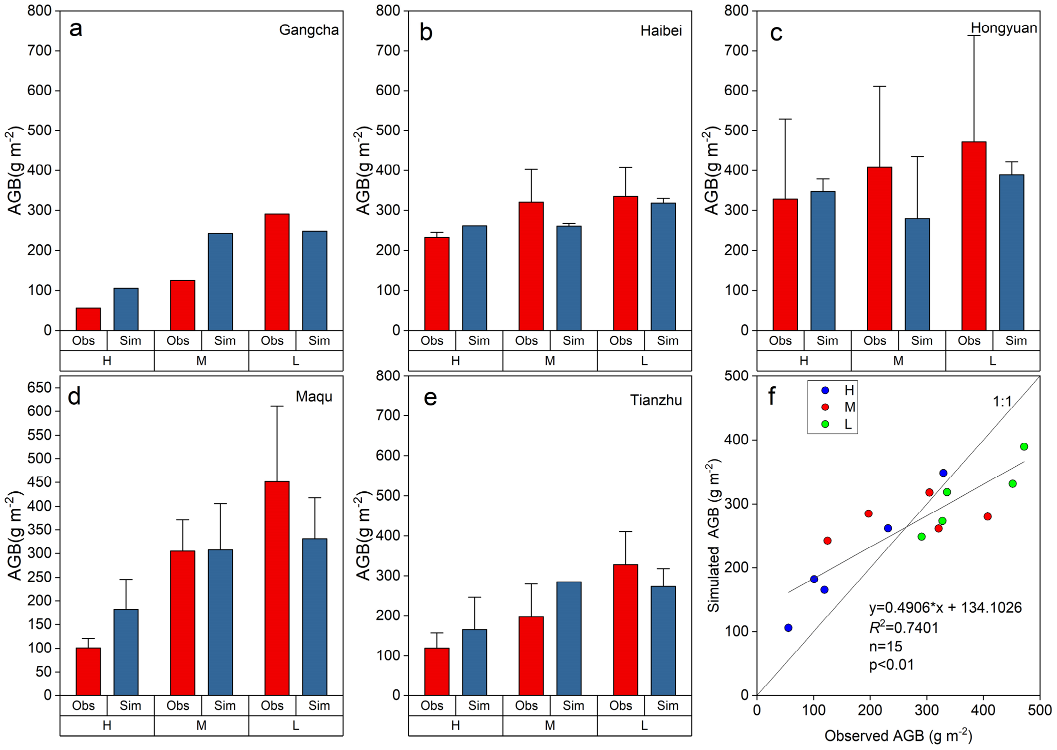

Supplementary Figure 7 Comparison between observed aboveground biomass (red) at experimental field sites and the corresponding simulated aboveground biomass (blue) on grasslands subject to light, medium or heavy stocking rate. Field sites were grouped into county clusters (see Supplementary Table 3 and Supplementary Figure 4) and error bars represent standard deviation (a-e). f: Comparison of mean observed and the corresponding simulated aboveground biomass for five county clusters subject to light, medium or heavy stocking rate based on panel a-e. AGB, above-ground biomass; H, heavy grazing; L, light grazing; M, medium grazing; Obs, observed; Sim, simulated.

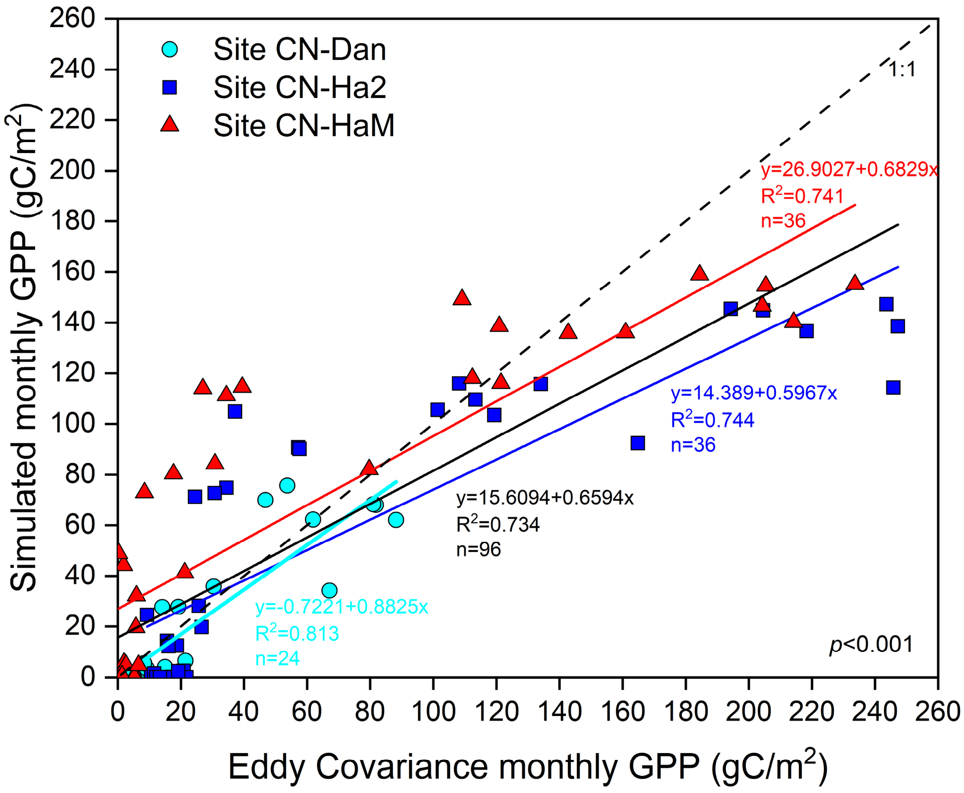

Supplementary Figure 8 Comparison between simulated monthly GPP and eddy covariance retrieved monthly gross primary productivity (GPP) at three FLUXNET sites (CN-Dan, CN-Ha2, CN-HaM) located on QTP. The data were available between 2004 and 2005, 2003 and 2005, 2002 and 2004 for site of CN-Dan, CN-Ha2, CN-HaM, respectively. The black solid line represented linear regression line for all the data records of the three sites.

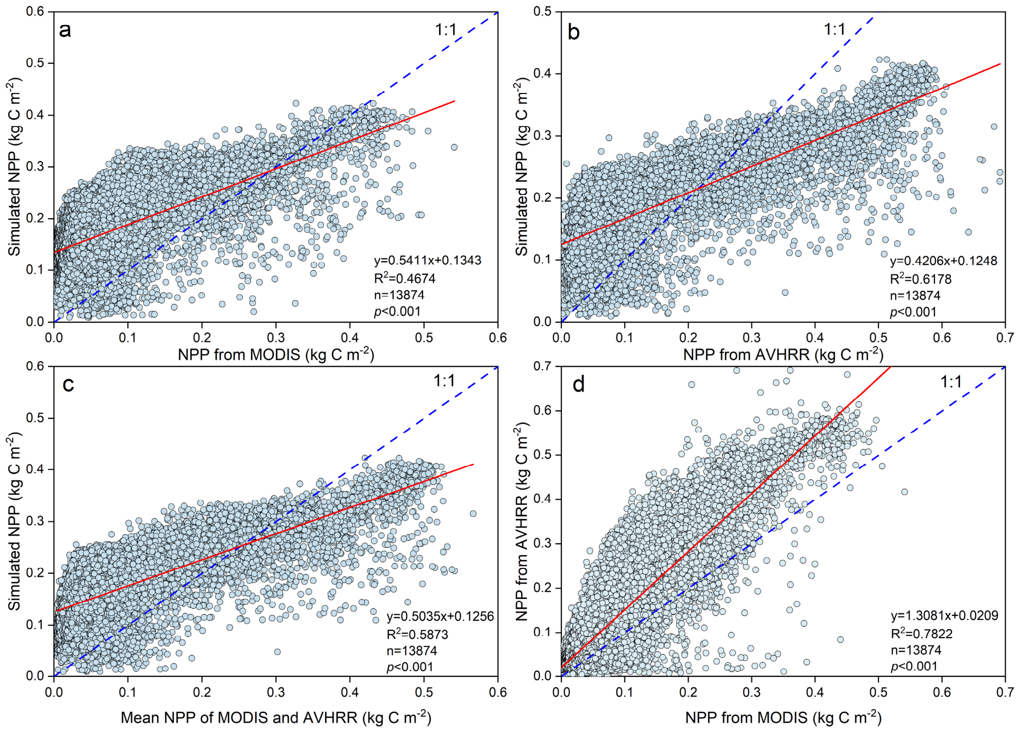

Supplementary Figure 9 Comparison at grid scale between simulated yearly net primary productivity (NPP) and multiyear average NPP across the Qinghai-Tibetan Plateau for the period 2000-2015 from remote sensing by (a) moderate-resolution imaging spectroradiometry (MODIS) or (b) high-resolution radiometry (AVHRR). (c) Simulated NPP was compared to the NPP averaged from MODIS and AVHRR measurements. (d) Comparison between NPP values from MODIS or AVHRR.

4. Supplementary Tables

Supplementary Table 1 Major parameters of plant functional types (PFT) of grasses

Plant function type	m	b	$V_{cmax,15}$	T_w(°C)	GDD_0	σ (m^2kg^{-1})	α_{leaf}	α_{root}	τ_{leaf} (years)	τ_{root} (years)
Warm (C4) grasses	4.0	0.04	4.0	>22.0	>100	20.0	0.45	0.55	1.25	1.00
Cool (C3) grasses	9.0	0.01	25.0	/	>100	20.0	0.45	0.55	1.00	1.00

Note: m, slope of stomatal conductance relationship (nondimensional); b, intercept of the stomatal conductance relationship (mol H_2O m^{-2} s^{-1}); $V_{cmax,15}$, maximum Rubisco capacity of the top leaf (μmol CO_2 m^{-2} s^{-1}) at 15°C; T_w, temperature of the warmest month; GDD_0, growing degree days calculated on a 0°C base; σ, the specific leaf area; α_{leaf} and α_{root}, the allocation fraction of total photosynthate to leaf and root; τ_{leaf} and τ_{root}, the residence time of carbon in leaf and root. The parameters of the two grass PFTs in the original IBIS model [37] were adjusted to fit the natural vegetation distribution in China [38].

Supplementary Table 2 Conversion coefficient of livestock units to sheep units (from Ren, 1998[39])

Livestock	Conversion coefficient to sheep unit (SU)
Sheep	1.0
Goat	0.9
Pig	1.0
Cattle	5.0
Yak	3.0
Horse/Mule	5.0
Donkey	3.5
Camel	7.0

Supplementary Table 3 Information about field sites from experimental grazing studies used in the present study to evaluate model performance. Data for aboveground biomass (AGB) were taken from sites marked with stars. Sites were grouped into ten county clusters (also see Supplementary Figure 4).

ID	Longitude	Latitude	Group setting		Designed SR at heavy level (SU ha^{-1} $year^{-1}$)	Simulated SR threshold (SU ha-1 year-1)	Data Source
			ID	County			
1*	102.9417	37.2167	G1	Tianzhu	8.57	4.00	Li et al., 2015[40]
2	102.9420	37.2170	G1		6.25	4.00	Miao, 2015[41]
3*	102.7250	37.2050	G1		5.82	5.50	Yang, 2017[42]
4	102.7250	37.2080	G1		4.98	5.50	Yang et al., 2016[43]
5*	102.7250	37.2083	G1		7.20	5.50	Niu et al., 2017[44]
6*	102.7250	37.2083	G1		7.20	5.50	Niu et al., 2018[45]
7	102.7750	37.1750	G1		6.00	5.00	Li, 2014[46]
8	102.7869	37.1769	G1		5.59	3.50	Zhou et al., 2008[47]
9	102.7869	37.1936	G1		3.75	3.50	Miao et al., 2014[48]
10	102.7833	37.1667	G1		3.75	3.50	Miao, 2012[49]
11	102.7869	37.1769	G1		3.75	3.50	Miao et al., 2016[50]
12*	101.1784	37.6665	G2	Haibei	9.25	6.00	Wang et al., 2008[51]
13*	101.1942	37.6722	G2		11.25	6.00	Dai et al., 2019[52]
14*	101.2917	37.6167	G2		15.00	6.00	Li et al., 2018[53]
15	101.2900	37.6150	G2		5.35	6.00	Wang, 2009[54]
16	101.2900	37.6150	G2		7.00	6.00	Li et al., 2014[55]
17	101.2917	37.6167	G2		5.35	6.00	Zhou et al., 2019[56]
18*	101.2500	37.5333	G2		5.35	5.00	Cao et al., 2003[57]
19	101.3750	37.6150	G2		5.83	6.00	Zhou et al., 2004[58]
20	101.4000	37.5833	G2		14.67	6.00	Ma et al., 2009[59]
21*	100.0667	37.3500	G3	Gangcha	5.62	5.00	Wang et al., 2016[60]
22	101.8833	33.9667	G4	Maqu	3.20	9.00	Tan, 2012[61]
23*	101.8830	33.9670	G4		7.70	9.00	Hu, 2015[62]
24	101.8833	33.9667	G4		16.00	9.00	Liu, 2018[63]
25*	102.1172	33.7058	G4		9.60	9.50	Li et al., 2011[64]
26*	101.7667	33.6667	G4		8.58	9.00	Zou et al., 2015[65]
27	99.7939	33.6225	G5	Guoluo	5.84	6.00	Dong et al., 2004[66]; Dong et al., 2012[67]
28	100.2392	34.4787	G5		6.24	7.00	Liu et al., 2005[68]
29	99.7800	33.6170	G5		5.01	6.00	Fu et al., 2013[69]
30	96.5000	33.2000	G6	Yushu	5.59	6.50	Zhou et al., 2008[47]

31	96.5167	33.1833	G6		7.47	6.50	Li et al., 2015[70]
32	93.6333	30.4500	G7	Linzhi	1.67	1.50	Yixi et al., 2014[71]
33	89.4250	33.0500	G8	Zangbei	5.99	3.50	Duan, 2011[72]
34	92.0014	31.4425	G9	Naqu	6.61	6.00	Wei et al., 2005[73]
35	102.6017	32.9172	G10	Hongyuan	4.80	9.00	Zheng et al., 2017[74]
36*	102.5830	33.0830	G10		14.55	9.50	Zhu et al., 2016[75]
37*	102.5500	32.8000	G10		9.00	9.00	Mipam et al., 2019[76]
38*	102.6000	33.0500	G10		8.70	9.50	Gao et al., 2008[77]

Supplementary Table 4 Overview of selected representative concentration pathways (RCPs)[78]

RCPs	Description
2.6	Peak in radiative forcing at ~3 W/m^2 (~490 ppm CO_2 eq) before 2100 and then decline (the selected pathway declines to 2.6 W/m^2 by 2100).
4.5	Stabilization without overshoot pathway to 4.5 W/m^2 (~650 ppm CO_2 eq) at stabilization after 2100
8.5	Rising radiative forcing pathway leading to 8.5 W/m^2 (~1370 ppm CO_2 eq) by 2100

Supplementary Table 5 List of all 24 General Circulation Models (GCMs) for future average climate calculation. Data source: https://esgf-node.llnl.gov/projects/cmip5/

No.	Model	Institution and country
1	BCC-CSM1-1	China Climate Center, China
2	BCC-CSM1-1-m	China Climate Center, China
3	BNU-ESM	Beijing Normal University, China
4	CanESM2	Canadian Centre for Climate Models and Analysis, China
5	CCSM4	National Center for Atmospheric Research (NCAR), USA
6	CESM1-CAM5	National Center for Atmospheric Research (NCAR), USA
7	CNRM-CM5	Centre National de Researches Meteorologiques, France
8	CSIRO-Mk3-6-0	CSIRO-Marine and Atmospheric Research, Australia
9	FGOALS-g2	State Key Laboratory for Numerical Simulation of Atmospheric Science and Hydrodynamics, Institute of Atmospheric Physics, Chinese Academy of Sciences, China
10	GFDL-CM3	Geophysical Fluid Dynamics Laboratory, USA
11	GFDL-ESM2G	Geophysical Fluid Dynamics Laboratory, USA
12	GFDL-ESM2M	Geophysical Fluid Dynamics Laboratory, USA
13	GISS-E2-H	Goddard Institute for Space Studies (NASA), USA
14	GISS-E2-R	Goddard Institute for Space Studies (NASA), USA
15	HadGEM2-AO	NIMR-KMA, South Korea
16	IPSL-CM5A-LR	Institut Pierre-Simon Laplace, France
17	IPSL-CM5A-MR	Institut Pierre-Simon Laplace, France
18	MIROC5	Atmosphere and Ocean Research Institute (University of Tokyo), Japan
19	MIROC-ESM	Japan Agency for Marine-Earth Science and Technology, Atmosphere and Ocean Research Institute (University of Tokyo), Japan
20	MIROC-ESM-CHEM	Japan Agency for Marine-Earth Science and Technology, Atmosphere and Ocean Research Institute (University of Tokyo), Japan
21	MPI-ESM-LR	Max-Planck Institute of Meteorology, Germany
22	MPI-ESM-MR	Max-Planck Institute of Meteorology, Germany
23	MRI-CGCM3	Meteorological Research Institute, Japan
24	NorESM1-M	Norwegian Climate Center, Norway

Nonendorsement disclaimer: Any use of trade, firm, or product names is for descriptive purposes only and does not imply endorsement by the U.S. Government.

Supplementary References

1. Cai HY, Yang XH, Xu XL. Human-induced grassland degradation/restoration in the central Tibetan Plateau: The effects of ecological protection and restoration projects. *Ecological Engineering* **83**, 112-119 (2015).

2. Dong QM, Zhao XQ, Wu GL, Shi JJ, Ren GH. A review of formation mechanism and restoration measures of "black-soil-type" degraded grassland in the Qinghai-Tibetan Plateau. *Environmental Earth Sciences* **70**, 2359-2370 (2013).

3. Feng RZ, Long RJ, Shang ZH, Ma YS, Dong SK, Wang YL. Establishment of Elymus natans improves soil quality of a heavily degraded alpine meadow in Qinghai-Tibetan Plateau, China. *Plant and Soil* **327**, 403-411 (2010).

4. Liu J, Xu X, Shao Q. The Spatial and Temporal Characteristics of Grassland Degradation in the Three-River Headwaters Region in Qinghai Province (in Chinese with English abstract). *Acta Geographica Sinica* **63**, 364-376 (2008).

5. Ma Y, Lang B. Review and Prospect of the Study on Black Soil Type Deteriorated Grassland (in Chinese with English abstract). *Pratacultural Science* **16**, 5-9 (1999).

6. Ren GH, Shang ZH, Long RJ, Hou Y, Deng B. The relationship of vegetation and soil differentiation during the formation of black-soil-type degraded meadows in the headwater of the Qinghai-Tibetan Plateau, China. *Environmental Earth Sciences* **69**, 235-245 (2013).

7. Sheehy D. The rangelands, land degradation and black beach: a review of research reports and discussions. In: *The Living Plateau:Changing Lives of Herders in Qinghai* (eds van Wageningen N, Sa W) (2001).

8. Wang XX*, et al.* A comparison of biodiversity-ecosystem function relationships in alpine grasslands across a degradation gradient on the Qinghai-Tibetan Plateau. *Rangeland Journal* **37**, 45-55 (2015).

9. Wu GL, Du GZ, Liu ZH, Thirgood S. Effect of fencing and grazing on a Kobresia-dominated meadow in the Qinghai-Tibetan Plateau. *Plant and Soil* **319**, 115-126 (2009).

10. Wu GL, Ren GH, Dong QM, Shi JJ, Wang YL. Above- and Belowground Response along Degradation Gradient in an Alpine Grassland of the Qinghai- Tibetan Plateau. *Clean-Soil Air Water* **42**, 319-323 (2014).

11. Cao JJ, Adamowski JF, Deo RC, Xu XY, Gong YF, Feng Q. Grassland Degradation on the Qinghai-Tibetan Plateau: Reevaluation of Causative Factors. *Rangeland Ecology & Management* **72**, 988-995 (2019).

12. Li XL, Gao J, Brierley G, Qiao YM, Zhang J, Yang YW. Rangeland degradation on the Qinghai-Tibet Plateau: Implications for rehabilitation. *Land Degradation & Development* **24**, 72-80 (2013).

13. Wang ZQ*, et al.* Quantitative assess the driving forces on the grassland degradation in the Qinghai-Tibet Plateau, in China. *Ecol Inform* **33**, 32-44 (2016).

14. Harris RB. Rangeland degradation on the Qinghai-Tibetan plateau: A review of the evidence of its magnitude and causes. *J Arid Environ* **74**, 1-12 (2010).

15. Berry L. Land Degradation in China: Its Extent and Impact. Land Degradation Case Studies – China.) (2003).

16. Hu ZZ, Zhang DG. China's pasture resources. In: *Transhumant Grazing Systems in Temperate Asia. Plant Production and Protection Series 31* (ed Suttie JM, Reynolds, S.B.). Food and Agriculture Organization of the United Nations (2003).

17. Liu YY, Wang Q, Zhang ZY, Tong LJ, Wang ZQ, Li JL. Grassland dynamics in responses to climate variation and human activities in China from 2000 to 2013. *Science of the Total Environment* **690**, 27-39 (2019).

18. Nan Z. The grassland farming system and sustainable agricultural development in China. *Grassland Science* **51**, 15-19 (2005).

19. Unkovich M, Nan Z. Problems and prospects of grassland agroecosystems in western China. *Agriculture Ecosystems & Environment* **124**, 1-2 (2008).

20. Wang XH, Han XY, Bennett J. Sustainable Land Use Change in the North West Provinces of China. Research Report 1, Australian Centre for International Agricultural Research (ACIAR) Project: ADP/2002/021, ISSN: 1449-7433. https://crawford.anu.edu.au/pdf/staff/jeff_bennett/china_land_use/research_report_9.pdf(accessed 20230418). (2004).

21. World Bank Group. China - Air, land, and water : environmental priorities for a new millennium (English).) (2001).

22. Lu XS, Fan JW, Liu JH. Grassland Resource Conservation Development Strategy In: *Chinese grassland sustainable development strategy (In Chinese)* (ed Du QL). Chinese Agricultural Press (2006).

23. Wang P, Lassoie JP, Morreale SJ, Dong S. A critical review of socioeconomic and natural factors in ecological degradation on the Qinghai-Tibetan Plateau, China. *The Rangeland Journal* **37**, 1-9 (2015).

24. Zhou W*, et al.* Grassland degradation remote sensing monitoring and driving factors quantitative assessment in China from 1982 to 2010. *Ecological Indicators* **83**, 303-313 (2017).

25. Zhou H*, et al.* Alpine Grassland Degradation and Its Restoration in the Qinghai–Tibet Plateau. *Grasses* **2**, 31-46 (2023).

26. Kwon H-Y, Nkonya E, Johnson T, Graw V, Kato E, Kihiu E. Global Estimates of the Impacts of Grassland Degradation on Livestock Productivity from 2001 to 2011. In: *Economics of Land Degradation and Improvement – A Global Assessment for Sustainable Development* (eds Nkonya E, Mirzabaev A, von Braun J). Springer International Publishing (2016).

27. Bardgett RD*, et al.* Combatting global grassland degradation. *Nat Rev Earth Env* **2**, 720-735 (2021).

28. Gang CC*, et al.* Quantitative assessment of the contributions of climate change and human activities on global grassland degradation. *Environmental Earth Sciences* **72**, 4273-4282 (2014).

29. Gibbs HK, Salmon JM. Mapping the world's degraded lands. *Appl Geogr* **57**, 12-21 (2015).

30. He Y, Zhou H, Zhao X, Lai D, Zhao J. Alpine Grassland Degradation and its Restoration on Qinghai-Tibet Plateau (in Chinese with English abstract). *Journal of Grassland And Forage Science* **29**, 1-9 (2008).

31. Foggin M, Smith AT. Rangeland Utilization and Biodiversity on the Alpine Grasslands of Qinghai Province, People's Republic of China. In: *Conserving China's Biodiversity (II)* (eds PETER JS, WANG S, XIE Y). China Environmental Science Press (1996).

32. Sun Y*, et al.* Grazing intensity and human activity intensity data sets on the Qinghai-Tibetan Plateau during 1990–2015. *Geosci Data J* **9**, 140-153 (2022).

33. Meng N*, et al.* A high-resolution gridded grazing dataset of grassland ecosystem on the Qinghai-Tibet Plateau in 1982-2015. *Sci Data* **10**, (2023).

34. Yang Z, Yang G. Potential productivity and livestock carrying capacity of high-frigid grassland in China (in Chinese with English abstract). *Resources Science* **22**, 72-77 (2000).

35. Qian S, Mao L, Hou Y, Fu Y, Zhang H, Du J. Livestock Carrying Capacity and Balance between Carrying Capacity of Grassland with added Forage and Actual Livestock in the Qinghai-Tibet Plateau (in Chinese with English abstract). *Journal of Natural Resources* **22**, 390-397 (2007).

36. Zhang YJ, Zhang XQ, Wang XY, Liu N, Kan HM. Establishing the carrying capacity of the grasslands of China: a review. *Rangeland Journal* **36**, 1-9 (2014).

37. Kucharik CJ*, et al.* Testing the performance of a Dynamic Global Ecosystem Model: Water balance, carbon balance, and vegetation structure. *Global Biogeochem Cy* **14**, 795-825 (2000).

38. Yuan Q, Zhao D, Wu S, Dai E. Validation of the Integrated Biosphere Simulator in simulating the potential natural vegetation map of China. *Ecological Research* **26**, 917-929 (2011).

39. Ren J. *Research Methods for Grassland Science*. China Agricultural Press (1998).

40. Li W*, et al.* Analysis of soil respiration under different grazing management patterns in the alpine meadow-steppe of the Qinghai-Tibet Plateau. *Acta Prataculturae Sinica* **24**, 22-32 (2015).

41. Miao F. Response of plant community to stocking rate and precipitation variation in the grassland of northeastern edge of Qinghai-Tibetan Plateau.). Lanzhou University (2015).

42. Yang S. Studies on the effects of short-term grazing on plant community and soil of alpine meadow.). Gansu Agricultural University (2017).

43. Yang S*, et al.* Effect of Short-term Grazing on Surface Soil Infiltration and Soil Water Retention in Alpine Meadow. *J Soil Water Conserv* **30**, 96-101 (2016).

44. Niu Y, Yang S, Wang G, Liu L, Du G, Hua L. Relation between species distribution of plant community and soil factors under grazing in alpine meadow. *Chinese journal of applied ecology* **28**, (2017).

45. Niu Y, Yang S, Wang G, Liu L, Du G, Hua L. Relationship between plant species, life form, functional group diversity, and biomass under grazing disturbance for four years on an alpine meadow. *Acta Ecologica Sinica* **38**, (2018).

46. Li S. Response of soil nutrients characteristics of alpine meadow to grazing density in the northeastern edge of Qinghai-Tibetan Plateau.). Lanzhou University (2014).

47. Zhou B, Li F, Yan L, Cairen Z, Geng X. Astudy on response of soil moisture of alpine wetland to grazing intensity. *Pratacultural Science*, 75-78 (2008).

48. Miao F, Li S, Xue R, Wang X, Guo Z, Shen Y. Response of forage quality of the dominant species to short-term grazing in the alpine meadow at Qinghai-Tibetan Plateau. *Pratacultural Science* **31**, 915-921 (2014).

49. Miao F. Response of community characteristics of alpine meadow to enclosure and grazing utilization in the alpine meadow to enclosure and grazing utilization in the Qinghai-Tibetan Plateau.). Lanzhou University (2012).

50. Miao F, Xue R, Guo Z, Shen Y. Influence of yak grazing on plant niche characteristics in alpine meadow communities at the northeastern edge of the Qinghai-Tibetan Plateau. *Acta Prataculturae Sinica* **25**, 88-97 (2016).

51. Wang C*, et al.* Vegetation roots and soil physical and chemical characteristic changes in Kobresia pygmaca meadow under different grazing gradients. *Acta Prataculturae Sinica*, 9-15 (2008).

52. Dai L*, et al.* Moderate grazing promotes the root biomass in Kobresia meadow on the northern Qinghai–Tibet Plateau. *Ecology and Evolution* **9**, (2019).

53. Li H, Wei Y, He H, Yang Y, Li Y. Effects of Grazing Density on Nitrous Oxide Effluxes in Alpine Kobresia Humilis Meadow on the Qinghai-Tibetan Plateau. *Chinese Journal of Agrometeorology* **39**, 27-33 (2018).

54. Wang W. Effects of different grazing patterns and habitat resources on grazing tolerance of two clonal plants in alpine meadow.). Shaanxi Normal University (2009).

55. Li B, Ge S, Xu T, Xu S, Li S. Effects of grazing intensity on net ecosystem exchange of alpine meadow. *Pratacultural Science* **31**, 1203-1210 (2014).

56. Zhou G*, et al.* Effects of grazing intensity on community structure and the soil's physical and chemical properties in an alpine meadow on the Eastern Qinghai-Tibet Plateau. *Pratacultural Science* **36**, 1022-1031+1918 (2019).

57. Cao G, Tang Y, Mo W, Wang Y, Li Y, Zhao X. Grazing intensity alters soil respiration in an alpine meadow on the Tibetan plateau. *Soil Biology and Biochemistry* **36**, (2003).

58. Zhou H, Zhao X, Tang Y, Zhou L, Liu W, Yu L. Effect of Long-term Grazing on Alpine Shrub Vegetation in Qinghai-Tibet Plateau. *Chinese Journal of Grassland*, 2-12 (2004).

59. Ma C*, et al.* Effects of Grazing Intensity on the Growth of Heifer Yak. *Journal of Domestic Animal Ecology* **30**, 40-43 (2009).

60. Wang J, Zhong M, Wu R, Dong Q, Wang K, Shao X. Response of plant functional traits to grazing for three dominant species in alpine steppe habitat of the Qinghai-Tibet Plateau, China. *Ecological research* **31**, (2016).

61. Tan Y. Research on the soil enzyme activities and soil nutrients in the alpine meadow of the

northeast Qinghai-Tibet Plateau.). Lanzhou University (2012).

62. Hu J. The studies on the responses of nematode communities on fertilization and grassing on eastern Tibetan plateau alpine meadows.). Lanzhou University (2015).

63. Liu Y. Greenhouse gas emissions of alpine meadow grazing systems on the Qinghai-Tibet Plateau.). Lanzhou University (2018).

64. Li W, Huang H, Zhang Z, Wu G. Effects of grazing on the soil properties and C and N storage in relation to biomass allocation in an alpine meadow. *Journal of soil science and plant nutrition* **11**, (2011).

65. Zou YL, Niu DC, Fu H, Zhang YC, Wan CG. Moderate grazing promotes ecosystem carbon sequestration in an alpine meadow on the Qinghai-Tibetan Plateau. *Journal of Animal and Plant Sciences-Japs* **25**, 165-171 (2015).

66. Dong Q, Li Q, Ma Y, Shi J. Effects of yaks stocking pates on aboveground and belowground blomass in kobrecia parva alpine meadow. *Pratacultural Science*, 48-53 (2004).

67. Dong Q*, et al.* Influence of grazing on biomass，growth ratio and compensatory effect of different plant groups in Kobresia parva meadow. *Acta Ecologica Sinica* **32**, 2640-2650 (2012).

68. Liu Y, Li Y, Lai D, Hu Z. Effect of grazing perennial artificial grassland in warm season on gain of Yak calves in the Alpine Meadow. *Grassland and Turf*, 53-57 (2005).

69. Fu J, Yixi C, Chen H, Miao Y, Hu T, Xu Y. Responses of dominant plant nutrients to grazing intensity in Kobresia pygmaea meadow of the Qinghai-Tibet plateau. *Pratacultural Science* **30**, 560-565 (2013).

70. Li F, Li X, Zhou B, Qi D, Wang L, Fu H. Effects of grazing intensity on biomass and soil physical and chemical characteristics in alpine meadow in the source of three rivers. *Pratacultural Science* **32**, 11-18 (2015).

71. Yixi C*, et al.* Effects of grazing intensity on vegetation community and soil physicochemical properties of alpine meadow in Tibet. *Journal of Northwest A & F University(Natural Science Edition)* **42**, 27-33 (2014).

72. Duan M. Remote Sensing Monitoring of Stipa Purpurea Alpine Grassland Aboveground Biomass under the Grazing Disturbance in Northern Tibet.). Chinese Academy of Agricultural Sciences (2011).

73. Wei X, Yang P, Li S, Chen H. Effects of over-grazing on vegetation degradation of the Kobresia pygmaea meadow and determination of degenerative index in the Naqu Prefecture

of Tibet. *Acta Prataculturae Sinica*, 41-49 (2005).

74. Zheng Q*, et al.* Effect of grazing intensity on species richness and biomass of alpine meadow in northwest Sichuan. *Pratacultural Science* **34**, 1390-1396 (2017).

75. Zhu E*, et al.* Early influence of the grazing intensity on ecosystem respiration of alpine meadows. *Chinese Journal of Applied and Environmental Biology* **22**, 561-566 (2016).

76. Mipam T, Zhong L, Liu J, Miehe G, Tian L. Productive Overcompensation of Alpine Meadows in Response to Yak Grazing in the Eastern Qinghai-Tibet Plateau. *Frontiers in Plant Science* **10**, (2019).

77. Gao YH, Luo P, Wu N, Chen H, Wang GX. Impacts of grazing intensity on nitrogen pools and nitrogen cycle in an alpine meadow on the eastern Tibetan plateau. *Applied Ecology and Environmental Research* **6**, 69-79 (2008).

78. van Vuuren DP*, et al.* The representative concentration pathways: an overview. *Climatic Change* **109**, 5-31 (2011).
