# Supplementary material for: An early warning signal for grassland degradation on the Qinghai-Tibetan Plateau
Source: Nat Commun. 2023 Oct 12;14:6406. doi: 10.1038/s41467-023-42099-4 (PMC10570289; doi:10.1038/s41467-023-42099-4)
Supplement: Supplementary file 3 — Reporting Summary [file 41467_2023_42099_MOESM3_ESM.pdf]

nature portfolio

Corresponding author(s): Qiuan ZHU; Yanfen WANG

Last updated by author(s): Sep 4, 2023

Reporting Summary

Nature Portfolio wishes to improve the reproducibility of the work that we publish. This form provides structure for consistency and transparency in reporting. For further information on Nature Portfolio policies, see our Editorial Policies and the Editorial Policy Checklist.

Statistics

For all statistical analyses, confirm that the following items are present in the figure legend, table legend, main text, or Methods section.

n/a	Confirmed	
☐	☒	The exact sample size (*n*) for each experimental group/condition, given as a discrete number and unit of measurement
☒	☐	A statement on whether measurements were taken from distinct samples or whether the same sample was measured repeatedly
☐	☒	The statistical test(s) used AND whether they are one- or two-sided<br>*Only common tests should be described solely by name; describe more complex techniques in the Methods section.*
☒	☐	A description of all covariates tested
☒	☐	A description of any assumptions or corrections, such as tests of normality and adjustment for multiple comparisons
☐	☒	A full description of the statistical parameters including central tendency (e.g. means) or other basic estimates (e.g. regression coefficient) AND variation (e.g. standard deviation) or associated estimates of uncertainty (e.g. confidence intervals)
☐	☒	For null hypothesis testing, the test statistic (e.g. *F*, *t*, *r*) with confidence intervals, effect sizes, degrees of freedom and *P* value noted<br>*Give P values as exact values whenever suitable.*
☒	☐	For Bayesian analysis, information on the choice of priors and Markov chain Monte Carlo settings
☒	☐	For hierarchical and complex designs, identification of the appropriate level for tests and full reporting of outcomes
☒	☐	Estimates of effect sizes (e.g. Cohen's *d*, Pearson's *r*), indicating how they were calculated

Our web collection on statistics for biologists contains articles on many of the points above.

Software and code

Policy information about availability of computer code

Data collection	No software was used in data collection. The original results were generated by a terrestrial ecosystem model and further results processing and analysis were conducted by R, ArcGIS, Originlab and Microsoft Excel. Custom R scripts for stocking rate detection and time until degradation detection were available at Figshare database (https://doi.org/10.6084/m9.figshare.23308475). The source code of the terrestrial ecosystem model of TRIPLEX-GHG is available by contacting the corresponding authors (zhuq@hhu.edu.cn; yfwang@ucas.ac.cn).
Data analysis	Custom R scripts for stocking rate threshold detection and time until degradation detection were constructed with R 4.1.0.. Spatial data processing and map creation in this study were conducted by ArcGIS (10.8). Other data analysis and figure creation in this study were conducted by Originlab 2021 and Microsoft Excel 2019.

For manuscripts utilizing custom algorithms or software that are central to the research but not yet described in published literature, software must be made available to editors and reviewers. We strongly encourage code deposition in a community repository (e.g. GitHub). See the Nature Portfolio guidelines for submitting code & software for further information.

Data

Policy information about availability of data

All manuscripts must include a data availability statement. This statement should provide the following information, where applicable:

- Accession codes, unique identifiers, or web links for publicly available datasets
- A description of any restrictions on data availability
- For clinical datasets or third party data, please ensure that the statement adheres to our policy

The source data underlying Figure1-6 are provided as Source Data files and have been deposited in the Figshare database (https://doi.org/10.6084/m9.figshare.23308475). Daily meteorological data of national meteorological stations were obtained from China Meteorological Data Service Centre (https://data.cma.cn/) and the interpolated meteorological driving data is publicly available at National Earth System Science Data Center (http://www.geodata.cn/). ESA CCI Land Cover time-series dataset can be obtained at http://maps.elie.ucl.ac.be/CCI/viewer/download.php. The monthly GPP dataset for three flux sites on QTP is publicly available at FLUXNET Network (https://fluxnet.org/data/download-data/). The NPP productions retrieved from Moderate Resolution Imaging Spectroradiometer (MODIS) is available at: http://files.ntsg.umt.edu/data/NTSG_Products/MOD17/GeoTIFF/MOD17A3/GeoTIFF_30arcsec/. The NPP productions retrieved from Advanced Very High Resolution Radiometer (AVHRR) is available at the link: http://www.glass.umd.edu/NPP/AVHRR/).

Research involving human participants, their data, or biological material

Policy information about studies with human participants or human data. See also policy information about sex, gender (identity/presentation), and sexual orientation and race, ethnicity and racism.

Reporting on sex and gender	This information has not been collected.
Reporting on race, ethnicity, or other socially relevant groupings	This information has not been collected.
Population characteristics	This information has not been collected.
Recruitment	This information has not been collected.
Ethics oversight	This information has not been collected.

Note that full information on the approval of the study protocol must also be provided in the manuscript.

Field-specific reporting

Please select the one below that is the best fit for your research. If you are not sure, read the appropriate sections before making your selection.

☐ Life sciences ☐ Behavioural & social sciences ☒ Ecological, evolutionary & environmental sciences

For a reference copy of the document with all sections, see nature.com/documents/nr-reporting-summary-flat.pdf

Ecological, evolutionary & environmental sciences study design

All studies must disclose on these points even when the disclosure is negative.

Study description	In this study, based on results of a terrestrial ecosystem model, we tried to detect a potential stocking rate threshold that could risk grassland extreme degradation on the QTP, apply the stocking rate threshold to identify grassland areas under threat of degradation, and predict when such degradation might occur under current conditions and future conditions of grazing, climate change and elevated CO2. We do not directly carry out any field measurement.
Research sample	For model performance evaluation, we collected monthly GPP data from three Fluxnet sites, which have 36, 36, 24 monthly records separately; We collected heavy stocking rate data from 38 experimental grazing studies and clustered the sites into 10 groups at county scale, in which 15 studies and 5 groups have above ground biomass data; We collected MODIS and AVHRR NPP data and have a comparison at grid scale with cell numbers of 13874. We do not directly carry out any field measurement.
Sampling strategy	For the model performance evaluation, we collected data from literatures or database as we can find. For the comparison between simulated NPP and remote sensed NPP, we only considered the grassland grid cell where the data are all available for simulated results, MODIS data and AVHRR data. We do not directly carry out any field measurement.
Data collection	Daily meteorological data of national meteorological stations were obtained from China Meteorological Data Service Centre (https://data.cma.cn/), the interpolated meteorological driving data is publicly available at National Earth System Science Data Center (http://www.geodata.cn/). For model performance evaluation, we collected data from published literatures (references were provided in supplementary information) and publicly opened dataset (FLUXNET, MODIS, AVHRR). Q.Z. and J.Z. recorded the data. We do not directly carry out any field measurement.
Timing and spatial scale	Meteorological driving data: from Jan-1960 to Dec-2017 over Qinghai-Tibetan Plateau; FLUXNET data: from Jan-2002 to Dec-2005 for three sites on Qinghai-Tibetan Plateau; Remote sensed NPP (MODIS and AVHRR) data: from Jan-2000 to Dec-2015 over Qinghai-

	Tibetan Plateau; Literatures collected data: published from 2003 to 2019 for 38 studies on Qinghai-Tibetan Plateau. We do not directly carry out any field measurement.
Data exclusions	For the comparison between simulated NPP and remote sensed NPP, we only considered the grassland grid cell where the data are all available for simulated results, MODIS data and AVHRR data. We do not directly carry out any field measurement.
Reproducibility	Data were collected from published literatures and the measurements are described in the references listed in supplementary materials. We do not directly carry out any field measurement.
Randomization	We do not directly carry out any field measurement.
Blinding	We do not directly carry out any field measurement.

Did the study involve field work? ☐ Yes ☒ No

Reporting for specific materials, systems and methods

We require information from authors about some types of materials, experimental systems and methods used in many studies. Here, indicate whether each material, system or method listed is relevant to your study. If you are not sure if a list item applies to your research, read the appropriate section before selecting a response.

Materials & experimental systems

n/a	Involved in the study	
☒	☐	Antibodies
☒	☐	Eukaryotic cell lines
☒	☐	Palaeontology and archaeology
☒	☐	Animals and other organisms
☒	☐	Clinical data
☒	☐	Dual use research of concern
☒	☐	Plants

Methods

n/a	Involved in the study	
☒	☐	ChIP-seq
☒	☐	Flow cytometry
☒	☐	MRI-based neuroimaging
